# Psychometric evaluation of the Decision Support System (DSS) for municipal nurses encountering health deterioration among older adults

**DOI:** 10.1186/s12877-024-04903-8

**Published:** 2024-03-26

**Authors:** Annica Kihlgren, Tomas Lammgård, Margaretha Norell Pejner, Fredrik Svensson, Ann-Sofie Adolfsson, Helen Lindner

**Affiliations:** 1 School of Health Sciences, Faculty of Medicine and Health, Örebro University, 701 82 Örebro, Sweden; 2 Older Adults’ Health and Living Condition, Örebro University, Örebro, Sweden; 3 Department of Home Care, Halmstad Municipality, Halmstad, Sweden

**Keywords:** Health deterioration, Older adult, Municipal registered nurse, Psychometric

## Abstract

**Background:**

A valid and reliable tool is crucial for municipal registered nurses (RNs) to make quick decisions in older adults who show rapid signs of health deterioration. The aim of this study was to investigate the psychometric properties of the Decision Support System (DSS) among older adults in the municipal healthcare system.

**Methods:**

Firstly, we utilized the Rasch dichotomous model to analyze the DSS assessments (*n*=281) that were collected from municipal RNs working with older adults in the municipal healthcare system. We examined the properties of the DSS in terms of its unidimensionality, item fit, and separation indices. Secondly, to investigate inter-rater agreement in using the DSS, four experienced municipal RNs used the DSS to assess 60 health deterioration scenarios presented by one human patient simulators. The 60 DSS assessments were then analyzed using the ICC (2,1), percentage agreement, and Cohen κ statistics.

**Results:**

The sample of older adults had a mean age of 82.8 (SD 11.7). The DSS met the criteria for unidimensionality, although two items did not meet the item fit statistics when all the DSS items were analyzed together. The person separation index was 0.47, indicating a limited level of separation among the sample. The item separation index was 11.43, suggesting that the DSS has good ability to discriminate between and separate the items. At the overall DSS level, inter-rater agreements were good according to the ICC. At the individual DSS item level, the percentage agreements were 75% or above, while the Cohen κ statistics ranged from 0.46 to 1.00.

**Conclusions:**

The Rasch analysis revealed that the psychometric properties of the instrument were acceptable, although further research with a larger sample size and more items is needed. The DSS has the potential to assist municipal RNs in making clinical decisions regarding health deterioration in older adults, thereby avoiding unnecessary emergency admission and helping to alleviate emergency department overcrowding.

**Supplementary Information:**

The online version contains supplementary material available at 10.1186/s12877-024-04903-8.

## Introduction

The increasing global prevalence of an aging population has led to a growing challenge for municipal healthcare services in Europe and worldwide [1]. Older adults prefer to live at home for as long as possible, and they often have common comorbid conditions, including heart disease, hypertension, respiratory disease, diabetes, joint disease, sensory impairment, and mental health problems [2]. The combination of different comorbid conditions in older adults makes it difficult to interpret rapid shifts in health statues. When community-dwelling older adults show rapid signs of clinical health deterioration, clinical decisions must be made quickly regarding whether the older adults are safe to continue staying at home or if they should go to the emergency room [3]. A reliable and valid instrument to support clinical decisions for health deterioration is thus crucial for municipal healthcare services.

Although clinical health deterioration is a common phenomenon among older adults, it is a challenge to interpret the symptoms for clinical health deterioration due to the lack of a consensus definition. Jones has proposed the following definition “*a deteriorating patient is one who moves from one clinical state to a worse clinical state which increases their individual risk of morbidity, including organ dysfunction, protracted hospital stay, disability, or death*” [4]. To assess the shift between clinical statues, the authors suggest healthcare professionals to take a person-centered perspective, which not only considers vital sign derangement but also factors such as old age, the number and extent of organ dysfunctions, and pre-morbid functional status.

Several scales have been developed with the aim of detecting signs of clinical health deterioration, either in a hospital ward or pre-hospital setting. Early Warning Scores (EWS) uses a scoring system to identify patients who are at risk of deteriorating. It assesses vital signs, level of consciousness, and other clinical parameters to generate a score [5]. In Sweden, a Rapid Emergency Triage and Treatment System is used at the emergency room [6, 7]. This process-based triage system classifies the patients based on the severity of vital signs together with medical history and underlying illnesses. A recent review has identified 14 decision support systems for prehospital emergency medical services [8], however, there is a lack of information regarding the psychometric properties of these 14 systems for community-dwelling older adults.

The Decision Support system (DSS) is a scale developed to investigate health deterioration in community-dwelling older adults who are registered in the home health care system [9]. It is intended for gathering health information to support municipal RNs in making decisions. The DSS takes a person-centered approach and is specifically developed and adapted for older adults. In addition to considering vital sign derangement, it also incorporates the evaluation of organ dysfunctions and pre-morbid functional status, including an assessment of exclusion symptoms, alongside the clinical judgement of RNs. The development of the DSS was first published in 2016 [9] and the result showed that 94 % of RN decisions to emergency room were ultimately hospitalized. The sensitivity and specificity were also within acceptable ranges. The RNs in the study described that the DSS provides decision support for them to work more systematically and to communicate more effectively with other healthcare services.

Although the previous findings of the DSS suggest that it is useful in assessing rapid shifts in health deterioration, it is clinically important to investigate its psychometric properties. For the DSS to function as a valid clinical decision support system, it is necessary to examine how vital parameters and exclusive symptoms work together to measure a single construct, which can be conceptualized as unidimensionality. Are there any DSS items that function differently than others? Furthermore, to what extent do different RNs, or raters, produce the same score when investigating the same health deterioration condition? Therefore, the aim of this study is to investigate the psychometric properties of the Decision Support System.

## Methods

### Study design

The methodological design consisted of two parts. In Part 1, we used Rasch model to perform a psychometric analysis of the data from the first DSS study in year 2016 [9]. In part 2, we investigated inter-rater agreement of DDS in year 2019, using simulated health deterioration scenarios.

### Ethical considerations

Part 1 of the study was ethically approved by the Uppsala Regional Ethical Review Board (registration number 2013/523). The data included 281 older persons who were assessed by the DSS during a two-week period in year 2016 in two municipalities.

### Setting

Part 1: The participants were older adults enrolled in the municipal home health care system, residing either in Nursing Homes or regular households. A Registered Nurse (RN), whether on the day or night shift, was notified by relatives, the patient directly, or through social services concerning potential declines in health. Subsequently, the RN visited the older individual, either at their residence or in the nursing home. During this visit, the RN utilized the DSS assessment to evaluate the health status of the older person when they exhibited sudden signs of health deterioration.

Part 2: In the municipal RN work routine, one RN visits an older person who is suspected of having a rapid or acute health deterioration. It is not common to have more than one RN (i.e., one rater) during the home visit. Between year 2019 and 2021, in order to investigate inter-rater agreements between RN, four RN by profession used the DSS to assess different health deterioration conditions (total 60 conditions). These conditions were derived from authentic patient cases in in the first DSS study [9] (See Supplement 1 for an example of a patient case).

**Fig. 1:**
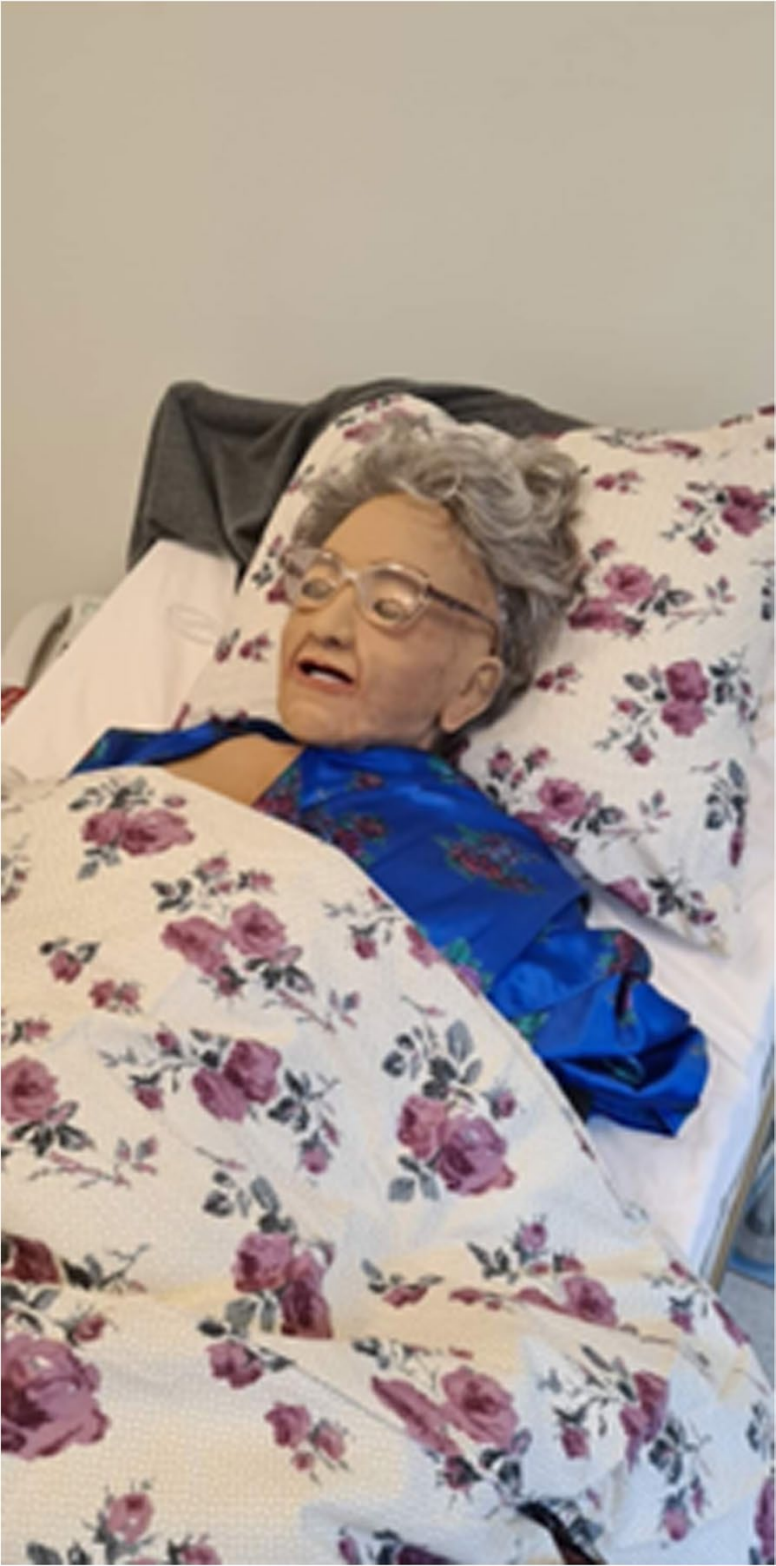
A human patient simulator at the clinical training center

A human patient simulator (HPS) (Fig. 1) was used to present 60 health deterioration scenarios for the investigation of inter-rater agreement. We used the Dieckmann model with seven phases (setting introduction, simulation briefing, theory input, scenario briefing, scenario, debriefing, ending) to prepare a full-scale simulation [10]. This was to prepare the raters (RNs) to understand the different health deterioration scenarios. The scenarios were designed to fit into a simulation environment. All 60 DSS assessments were performed in the same room.

As a high-fidelity level is essential in simulation-based scenarios [11] , we strived to re-create the health scenarios to mimic real-life situations as much as possible. The HPS was located in a furnished apartment at the clinical training center of our university. The apartment is equipped with an emergency bag with blood pressure manchet, stethoscopes, saturation meters and other standard equipment for assessing the medical condition of patients.

### The Decision-Support System (DSS)

The DSS is a clinical decision support system designed with a list of vital parameters and exclusion symptoms, along with the RN's own clinical judgment, to determine whether older persons are safe to continue staying at home or if they should go to the emergency room.

In the first step, the RN registers the patient’s ID and determines whether there is a current decision about palliative care with adequate prescriptions for symptom relief at home. If the answer is yes, no assessment of vital parameters is needed. The decision would be that the patient should stay at home and contact should be made with the responsible physician if needed. If the answer is no, the second step is to check the following vital parameters: free airways, breathing frequency within 8–25/min, saturation above or equal to 92%, heart rate, blood pressure, degree of consciousness, and temperature.

Then, in the third step, the RN investigates the existence of exclusion symptoms for being cared for at home. These include, for example, abdominal pain in relation to the use of a urinary catheter, dizziness, breathing problems, chest pain, diabetes, fever, affected general condition, and back pain. In the fourth step, the RN decides if the vital parameters are within the reference values or if there are any exclusion symptoms. The RN fills in the DSS and chooses between a green and a red box. The green box indicates that the older person can continue staying at home, while the red box indicates that the older person should go to the emergency room.

### Data collection procedure

Part 1: A RN, whether on the day or night shift, was notified by relatives, the patient directly, or through social services concerning rapid signs of health deterioration. Subsequently, the RN visited the older individual, either at their residence or in the nursing home. The RN followed the four steps in the DSS as described above. After the DSS was conducted, the result was sent to a responsible RN in each municipality and then to one of the authors (FS).

Part 2: Four RNs with more than 10 years of experience with health deterioration among municipal living older adults participated. Using the DSS, they assessed the health deterioration condition of the HPS. Raters 1 and 2 assessed deterioration scenarios 1-20 (*n*=20) whilst raters 3 and 4 assessed 21-60 (*n*=40) health deterioration scenarios. Author TL was in the room all the time to be able to provide support to what the simulator could not produce e.g., paleness or cold sweating. TL also acted as the patient's voice.

### Data analysis

#### Part 1: Psychometric testing using Rasch model

The DSS dichotomous data (281 assessments) were analyzed with the dichotomous Rasch model using Winsteps 5.3.2.0 [12]. It is a probabilistic model that estimates the probability of a positive response to an item depends on a person parameter and an item parameter in such a way that the probability of a positive response to an item depends on the product of the person parameter and the item parameter [13]. Rasch model focuses on the interaction of a person with an item rather than upon a total score in Classic Test Theory. During the analysis, the Rasch model converts dichotomous data (yes/no as 1/0) into interval logit measures, giving each item in the DSS a logit measure (Log-Odds Unit). The analysis validates an assessment by examining different characteristics of an assessment using different metrics and criteria. The sample size required to achieve stable item calibrations, i.e. an accuracy of ± 0.5 logits at a 95% confidence interval (CI), ranges from 64 to 144 subjects [14]. The sample size in the present study is thus enough to achieve stable item calibrations.

*Unidimensionality* refers to whether the questions in an assessment measure the same attribute. In the DSS, we investigated whether the DSS items work together to serve the underlying latent attribute “decision support for health deterioration”. Principal components analysis of residuals was used to examine whether the vital parameters and exclusion symptoms in the DSS work together to measure “decision support for health deterioration”. To fulfil the criteria for unidimensionality in Rasch, the raw variance explained by the measures should be above 60% and the unexplained variance in first contrast should be below 5%.

*Item fit statistics* examine the extent to which the observed data of each DSS item matches the one expected by the model. Each item produces infit and outfit normalized mean square (MnSq) residuals. Infit is an information-weighted MnSq statistic whereas outfit is an unweighted MnSq statistic. MnSq fit statistics show the size of the randomness, and the significance of MnSq is reported by Z-standardized (Zstd). For this study, an item with MnSq value greater than 1.4 or a Zstd value greater than 2.0 indicates a misfit, which means that the item’s performance does not match the expectations of the Rasch model. Infit values of less than 0.6 associated with a Zstd value of -2 suggest that an item is not contributing independent information. We performed the fit statistics analysis by first analyzing all the DSS items, i.e., the set of vital parameters and the set of exclusion parameters. If any item was misfit in the first analysis, we then analyzed separately the set of vital parameters and the set of exclusion parameters.

*Point–measure correlation of each item* reports the relationship between the group’s performance on the item and the group’s performance on the whole instrument. All items are expected to correlate positively in the direction of the latent variable, if any items show negative correlations, it is assumed that these items are considered invalid.

*Local item independence* assesses whether responses to any item are unrelated to any other item when trait level was controlled; thus, the endorsement of any item should not affect the probability of endorsement of the other items. Violation of local item independence may affect parameter estimates. An item residual correlation of at least 0.7 (i.e., common variance approximately 0.50) was set as a criterion for item dependency.

### Separation and reliability

Person separation indicates how well a set of items can separate those persons measured. Item separation indicates how well a sample of adults can separate those items used in the test. The separation index of ≥ 2.0 with a reliability of ≥ 0.80 is recommended.

#### Part 2: rater agreement

The ICC ^2,1^ (a two-way random effect model) was used to examine interrater agreements between the measurements of each pair of raters (rater 1 and 2, rater 3 and 4). An ICC > 0.70 is high for research purposes, and > 0.90 is necessary for clinical purposes [15]. Percentage agreement and Cohen κ statistics were used to examine inter-rater agreement at the item level.

## Results

### Part 1: results of rasch analysis

This sample of older persons (*n*=281) had a mean age of 82.8 (SD 11.7). Sixty-six females took part in the study (24 %) and 49 males (17 %). no information was available on sex for 166 participants (59 %). Because of health deterioration they all resided in their own homes or nursing homes during the time of the assessment.

#### Unidimensionality

The principal components analysis of all DSS items showed that the DSS fulfilled the criteria for unidimensionality. The raw variance explained by the measures (which should be above 60%) is 71% and the unexplained variance in first contrast (which should be below 5%) is 4.3%.

#### Item fit statistics

With the whole DSS, i.e., all the vital parameters and exclusion symptoms, the logit measures range from -5.33 (free airways, sum raw score=263) to 2.75 (back pain, sum raw score=17). Both Infit MnSq and Outfit MnSq of the DSS items are within the recommended criteria except for three exclusive symptoms “*breathing problem*”, “*chest pain*” and “Fever” (Table 1). The Outfit MnSq and Zstd of these three items are larger than 2.0.

**Table 1:** Item raw score, item logit measures and item fit statistics for vital parameters and exclusion symptoms

Items*	Total raw score**	Item logit measure	Infit MnSq***	Infit Zstd****	Outfit MnSq	Outfit Zstd
Free airways	262	-5.33	0.97	0.06	0.62	-0.38
Breathing frequency within 8–25/min	210	-2.16	1.02	.026	1.31	1.62
Saturation above or equal to 92 %	206	-2.04	0.98	-0.14	1.33	1.81
Heart rate between 50-100 s/min	202	-1.89	0.79	2.32	0.71	-2.04
Blood pressure (≥100 mm Hg)	242	-3.68	0.94	-0.24	1.28	0.77
Degree of conciousness	222	-2.81	0.92	-0.56	0.83	-0.59
Temperature	221	-3.09	0.92	-0.45	0.74	-0.81
Urinary catheter	14	2.97	0.97	-0.04	1.40	0.97
Dizziness	20	2.56	0.91	-0.47	1.27	0.77
Breathing problem	18	2.68	1.08	0.45	**2.55**	**2.93**
Chest pain	26	2.22	1.01	0.14	**2.37**	**3.03**
Diabetes	12	3.14	1.00	0.08	1.18	0.52
Fever (e.g. chill)	16	2.82	1.02	0.16	**2.24**	**2.41**
Affected general health	35	1.87	1.04	0.34	1.72	2.10
Back pain	17	2.75	0.96	-0.15	0 .67	-0.81

Items*: the items are presented in the same order as in the Decision Support System

Total raw score**: sum score of each item (Yes=1, No=0)

MnSq***= Mean Square standardized residuals, Zstd****= standardized Z-values

When analyzed only the vital parameters, both the Infit MnSqs and Outfit MnSqs are within the recommended criteria (Table 2). Similarly, when analyzed only the exclusive symptoms, the Infit MnSqs and Outfit MnSqs are within the recommended criteria (Table 3).

**Table 2:** Item raw score, item logit measures and item fit statistics for vita parameters in the Decision Support System

Items*	Total raw score**	Item logit measure	Infit MnSq***	Infit Zstd****	Outfit MnSq	Outfit Zstd
Free airways	262	-2.57	0.89	-0.15|	1.21	0.52
Breathing frequency within 8–25/min	210	0.95	1.04	0.49	1.26	**2.26**
Saturation above or equal to 92 %	206	1.14	0.97	-0.37	0.95	-0.47
Heart rate	202	1.30	0.83	-2.31	0.77	**-2.20**
Blood pressure	242	-0.86	0.96	-0.19	1.05	0.26
Degree of conciousness	222	0.18	1.14	1.17	1.30	1.70
Temperature	221	-0.14	1.03	0.24	1.04	0.24

Items*: the items are presented in the same order as in the Decision Support System

Total raw score**= total sum score of each item (Yes=1, No=0)

MnSq***=Mean Square standardized residuals, Zstd****= standardized Z-values

**Table 3:** Item raw score, item logit measures and item fit statistics for exclusion symptoms in the Decision Support System

Items*	Total raw score**	Item logit measure	Infit MnSq***	Infit Zstd****	Outfit MnSq	Outfit Zstd
Urinary catheter	14	0.38	1.03	0.23	1.08	0.38
Dizziness	20	-0.07	1.01	0.14	1.02	0.18
Breathing problem	18	0.07	1.11	0.68	1.20	0.96
Chest pain	26	-0.45	0.96	-0.28	0.95	-0.33
Diabetes	12	0.57	1.07	0.37	1.11	0.46
Fever	16	0.22	0.95	-0.19	0.93	-0.24
Affected general health	35	-0.87	0.98	-0.19	0.98	-0.19
Back pain	17	0.14	0.89	-0.56	0.79	-0.95

Items*: the items are presented in the same order as in the Decision Support System

Total raw score**: sum score of each item (Yes=1, No=0)

MnSq***= Mean Square standardized residuals, Zstd****= standardized Z-values

#### Point–measure correlations and local item independence

The point-measure correlations of the DSS items are between 0.05 and 0.62, which indicates that the items correlate positively in the direction of the latent variable. All items show standardized residual correlations below 0.7. The greatest standardized residual correlations are between item dizziness and degree of consciousness (0.42).

#### Separation indices

The person separation index is 0.47, which indicates that there is a restricted level of separation among the included participants. The item separation index is 11.43, which indicates that the DDS has a good ability to discriminate between and separate the items.

**Fig. 2:**
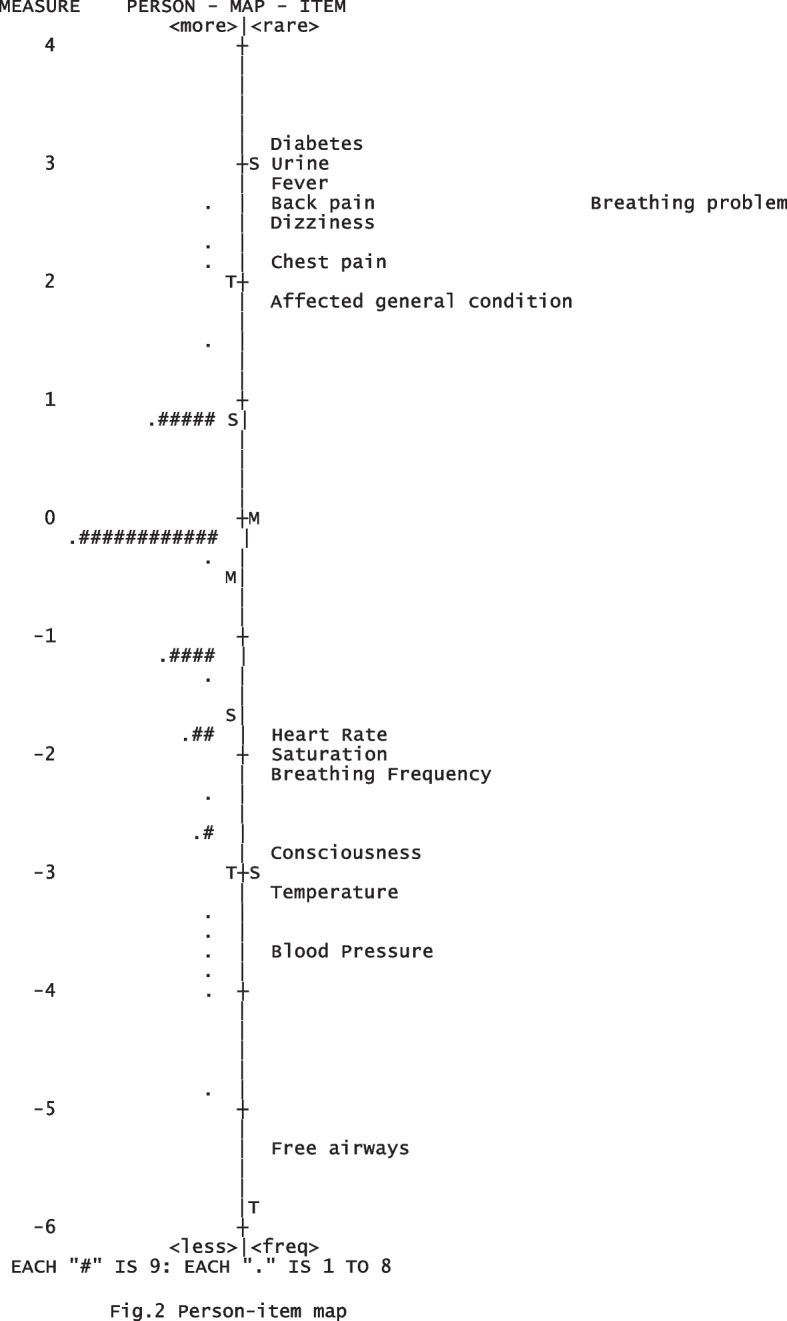
Person-item map

The person-item map visualizes how the items and participants fit together on a continuum. The mean of the participants is -0.45 and the mean of the DSS item is 0.00 by default. This indicates that the test-item targeting is satisfactory (Fig. 2). There is, however, a larger gap between the item “heart rate” and the item “affected health conditions”.

### Part 2: rater agreement

#### At the DSS assessment level

The ICC^2,1^ for 20 DSS assessments performed by raters 1 and 2 is 0.866 (CI 0.67-0.94, p) and the ICC ^2,1^ for the 40 assessments raters 3 and 4 is 0.828 (CI 0.68-0.91), indicating that the inter-rater reliability of both pairs of raters is good.

#### At the DSS item level

For raters 1 and 2, the percentage agreements of DSS items are between 76.3% and 100% whereas the Cohen κ are between 0.63 and 1. For raters 3 and 4, the percentage agreement of DSS items are between 75% and 100% whereas the Cohen κ are between 0.46 and 1.00 (Table 4).

**Table 4:** Rater agreements in percentage agreement and Cohen κ for the Decision Support System

	Rater 1 and 2 (20 patient cases)		Rater 3 and 4 (40 patient cases)	
Items*	PA(%)	Cohen κ(95% CI)	PA (%)	Cohen κ(95% CI)
Free airways	100	- (-)	100	1.00 (-)
Breathing frequency within 8–25/min	100	1.00 (-)	100	1.00 (-)
Saturation above or equal to 92 %	100	1.00 (-)	100	1.00 (-)
Heart rate between 50-100 s/min	100	1.00 (-)	100	1.00 (-)
Blood pressure (≥100 mm Hg)	100	1.00 (-)	100	1.00 (-)
Degree of conciousness (Reaction level scale is 1)	100	1.00 (-)	100	1.00 (-)
Temperature	100	1.00 (-)	100	1.00 (-)
Urinary catheter	95.0	0.65 (0.01,1.28)	90.0	0.62 (0.15,1.08)
Dizziness	95.0	-	75.0	0.50 (0.14.0.86)
Breathing problem	90.0	0.69 (0.28, 1.08)	85.0	0.70 (0.41.0.92)
Chest pain	100	1.00(-)	80.0	0.53 (0.14,0.92)
Diabetes	100	1.00(-)	100	1.00(-)
Fever symptom	95.0	0.78 (0.34, 1.20)	90.0	0.62 (0.14,1.08)
Affected general health	76.0	0.63(0.29, 0.99)	76.5	0.53 (0.12, 0.93)
Back pain	100	1.00 (-)	90.0	0.46 (-0.13, 1.05)

## Discussion

In the study we investigated the psychometric properties of the DSS using the Rasch dichotomous model and reliability statistics. The DSS fulfilled the criteria for unidimensionality, but not all items met the item fit statistics when analyzed together. At the overall DSS level, there were good rater agreements according to the ICC. At the individual DSS item level, the percentage agreements were 75% or above, but the Cohen values ranged from 0.46 to 100%.

The DDS has not undergone rigorous psychometric analysis before. It consists of two major parts: vital parameters and exclusive symptoms. The finding of unidimensionality is the first evidence that the DDS can be used as a single construct for assessing health deterioration. In an instrument that uses physical and cognitive functions to measure global functioning in older adults, their finding using Rasch also shows a single dimension with 54.2% variance [16]. Although a larger sample is needed to further validate this finding, the fact that the DDS is unidimensional suggests that both parts of the DDS can be used together to assess health deterioration and support clinical decision-making.

Three items did not fulfill the item fit statistics when all the DSS items were analyzed together. The Outfit MnSq and Zstd of the items "Breathing problem," "Chest pain," and "Fever" were larger than 2.0. However, the Infit MnSqs of all three items were smaller than 2.0, which is within the recommended range. Infit statistics are sensitive to unexpected responses close to an item measure, whereas Outfit statistics are sensitive to unexpected responses far from an item’s measure [13]. Therefore, this finding is not a significant threat to the overall validity of the DSS. When the analysis was performed only on the exclusive symptoms, the MnSq of both items was smaller than 2.0. This suggests that they functioned well within the exclusive symptoms. Further validation with a larger sample is needed to investigate whether these three items would continue to function differently when all the DSS items are analyzed together.

The person separation index is low, and this is because all the older adults in the sample have experienced a decline in health. This can also be observed in the person-item map, where the older adults are located or clustered in the middle part of the map. Furthermore, the person-item map indicates a gap between the items "heart rate" and "affected health conditions." During the development of the DSS, different experts and municipal RNs discussed whether additional items are necessary. One potential item for exclusion is "worry," as it is often a sign of rapid deterioration in health, such as heart problems or infections. Further research is needed to determine whether the DSS should include additional items, such as worry.

The results of the rater agreement serve an important purpose as they indicate the extent to which the DSS items are well understood. The percentage agreements were at least 75% for both the vital parameters and the exclusive symptoms. However, the Cohen κ coefficients ranged from 0.46 to 100%, despite the relatively high percentage agreements. This paradox is likely due to the effect of the level of chance agreement between the raters [17].

The findings of the study have two implications for healthcare systems. First, for municipal healthcare services, there is now a tool that has been psychometrically tested for community-dwelling older adults showing rapid shifts in health deterioration. By using the DSS, the health status of older adults can be ensured, facilitating nurses in making prompt clinical decisions for older adults to receive the most appropriate care. This may include decisions such as whether to continue staying at home or proceed to the emergency room. The DSS also provides reassurance to the individual nurse in their decision-making process. From an emergency care perspective, the psychometrically tested DSS may help municipal RNs ease emergency department crowding, which is a known and recognized problem [18].

### Method considerations

The results of the rater agreement in the study are interesting on several levels. Analysis using Cohen κ coefficients is an appropriate way of determining rater agreement but there are some shortcomings in the methodology which should be considered [19]. Care should be taken in generalizing these results to RNs who do not have clinical experience with health deterioration. It must be remembered that the Kappa analysis is based upon the assumption that the raters being measured are equally skilled [20].

Inter-rater agreement of the DSS was performed on the HPS using health deterioration scenarios from Part 1. This is a limitation because the DSS is intended for use during home visits in situations where older adults experience rapid shifts towards health deterioration. However, HPS is a valuable research tool for allows us to create realistic scenarios that mimic real-life medical situations, providing a clinical environment for investigating different health issues [21]. The use of HPS offers us both a safe and ethical way to investigate different levels of health deterioration.

For the statistical analysis in future studies, it would therefore be advantageous if the participants had similar experience and professional characteristics. Independent factors such as their level of training and their experience could also affect the measured degree of agreement between them so such factors should also be accounted for. Apart from the raters’ personal characteristics, results in this study could be affected by various procedural factors, such as how precisely the various deterioration scenarios to be rated are defined and described. In future studies it would be useful to be able to take into account the experience of the raters and the degree to which they interpret their task in a similar manner.

## Conclusion

Despite improvements in healthcare for older adults, there is a lack of clinical decision support systems that are adapted to their needs. This study reports the first psychometric testing of the DSS, and the results are promising. Further research and statistical analysis with larger samples and more items is needed to refine the DSS.

### Supplementary Information

**Supplementary Material 1.** 

## Data Availability

The datasets used and/or analysed during the current study are available from the corresponding author upon reasonable request.
