# Correction: Psychometric evaluation of the Decision Support System (DSS) for municipal nurses encountering health deterioration among older adults

**DOI:** 10.1186/s12877-024-04962-x

**Published:** 2024-05-13

**Authors:** Annica Kihlgren, Tomas Lammgård, Margaretha Norell Pejner, Fredrik Svensson, Ann-Sofie Adolfsson, Helen Lindner

**Affiliations:** 1 School of Health Sciences, Faculty of Medicine and Health, Örebro University, 701 82 Örebro, Sweden; 2 Older Adults’ Health and Living Condition, Örebro University, Örebro, Sweden; 3 Department of Home Care, Halmstad Municipality, Halmstad, Sweden

**Correction: BMC Geriatrics (2024) 24:283**

10.1186/s12877-024-04903-8

After publication of this article, the authors reported that the last part of the last sentence in the abstract, section ‘Conclusions’ ‘thereby avoiding unnecessary emergency admistion and helping.’ should have read ‘thereby avoiding unnecessary emergency admission and helping to alleviate emergency department overcrowding.’

The original article has been updated by the authors.

