# Supplementary material for: Psychometric evaluation of the Decision Support System (DSS) for municipal nurses encountering health deterioration among older adults
Source: BMC Geriatr. 2024 Mar 26;24:283. doi: 10.1186/s12877-024-04903-8 (PMC10964528; doi:10.1186/s12877-024-04903-8)
Supplement: Supplementary file 1 — Supplementary Material 1. [file 12877_2024_4903_MOESM1_ESM.pdf]

An example of one patient case

Main complaint: At time 13:40, the patient experienced stomach pain, chills, and fever.

Situation: During the afternoon, you receive a call from a nursing assistant who is at Edvin's, an 89-year-old individual, home. The nursing assistant Lena, contacting you, knows Edvin well and requests your visit to Edvin's home as something seems amiss. Lena notes that Edvin doesn't seem quite himself, and she is concerned about his restlessness and peculiar breathing.

Background: Edvin has a background as a farmer, which has been his lifelong profession. He lives with his wife on their farm. Edvin receives home care, assisting him with medications, and he also has a wound on his right foot that requires dressing every other day.

Edvin has early-stage vascular dementia, a history of a previous stroke, and prostate cancer.

Current situation: Upon meeting Edvin at his home, he expresses discomfort, complaining of stomach pain and feeling cold. You also observe that he is somewhat short of breath.

Decision for palliative care: NO

Vital parameters

- No known allergy
- Airway clear? Yes
- Respiratory rate: 36/min, regular
- Saturation: 88%
- Heart rate: 117 beats/min, regular rhythm
- Blood pressure: 110 mmHg, slightly difficult to obtain as the patient is not fully cooperative.
- Level of consciousness: RLS 2
- Temperature: 37.9 degrees

Decision Support System -outcomes:

A	Airway clear?	yes	Green
B	Respiratory rate	36	Red
B	Saturation	88	Red
C	Heart rate	117	Red
C	Blood pressure	110	Green
D	Level of consciousness	2	Red
E	Temperature	37,9	Green

Exclusion symptoms: Abdominal pain, episodes of chills, signs of infection, and severe pain.
